# Effects of Fluoroquinolones and Azithromycin on Biofilm Formation of *Stenotrophomonas maltophilia*

**DOI:** 10.1038/srep29701

**Published:** 2016-07-13

**Authors:** Aihua Wang, Qinqin Wang, Timothy Kudinha, Shunian Xiao, Chao Zhuo

**Affiliations:** 1 State Key Laboratory of Respiratory Diseases, the First Affiliated Hospital of Guangzhou Medical University, Guangzhou, China; 2 Department of Respiratory Medicine, the First Affiliated Hospital of Jinan University, Guangzhou, Guangdong, China; 3 Centre for Infectious Diseases and Microbiology Services, ICPMR-Pathology West, Westmead Hospital, University of Sydney, Darcy Road, New South Wales, Australia

## Abstract

*Stenotrophomonas maltophilia* is an opportunistic pathogen that causes respiratory and urinary tract infections, as well as wound infections in immunocompromised patients. This pathogen is difficult to treat due to increased resistance to many antimicrobial agents. We investigated the *in vitro* biofilm formation of *S. maltophilia*, including effects of fluoroquinolones (FQs) and azithromycin on biofilm formation. The organism initiated attachment to polystyrene surfaces after a 4 h incubation period, and reached maximal growth at 18–24 h. In the presence of FQs (moxifloxacin, levofloxacin or ciprofloxacin), the biofilm biomass was significantly reduced (*P* < 0.05). A lower concentration of moxifloxacin (10 μg/mL) exhibited a better inhibiting effect on biofilm formation than 100 μg/mL (P < 0.01), but with no difference in effect compared to the 50 μg/mL concentration (P > 0.05). However, the inhibitory effects of 10 μg/mL of levofloxacin or ciprofloxacin were slightly less pronounced than those of the higher concentrations. A combination of azithromycin and FQs significantly reduced the biofilm inhibiting effect on *S. maltophilia* preformed biofilms compared to azithromycin or FQs alone. We conclude that early use of clinically acceptable concentrations of FQs, especially moxifloxacin (10 μg/mL), may possibly inhibit biofilm formation by *S. maltophilia.* Our study provides an experimental basis for a possible optimal treatment strategy for *S. maltophilia* biofilm-related infections.

*Stenotrophomonas maltophilia* (*S. maltophilia*) is an opportunistic pathogen that is widely distributed in nature. It is detected in rivers, sewage, as well as in pharyngeal, sputum, and fecal samples of healthy individuals^1^,^2^. *S. maltophilia* often causes respiratory and urinary tract infections in immunocompromised patients. This pathogen, which is the third most common infectious non-fermentative bacterium after *Pseudomonas aeruginosa* and *Acinetobacter baumannii*^3^,^4^,^5^, can also cause severe bacteremia and endocarditis^2^,^6^. Risk factors for *S. maltophilia* infections include immune dysfunction and invasive clinical treatments to combat diseases, including endotracheal intubation and malignant tumors^2^,^7^,^8^.

A bacterial biofilm refers to a group of bacterial cells that adhere to one another on a surface. These adherent cells are often embedded within a self-produced matrix of an extracellular polymeric substance. Many infectious bacteria, such as *S. maltophilia*, *Pseudomonas aeruginosa* and *Staphylococcus aureus*, are capable of forming biofilms^9^. Biofilms exhibit greater resistance to antimicrobial drugs than non-biofilm forming bacteria, and are therefore more difficult to treat clinically^10^,^11^. It has been reported that high concentrations (500 μg/mL) of moxifloxacin, levofloxacin, or ciprofloxacin can significantly inhibit *S. maltophilia* biofilm formation, and that the inhibition is significantly reduced when the concentration is lowered to 50 μg/mL^12^. Treatment of biofilm-related infections using a combination of fluoroquinolone (FQ) and azithromycin has also been reported and appear to have different efficacies for different bacteria; the effect is synergetic for *Pseudomonas aeruginosa* and *Legionella* but antergic for *Mycobacterium avium* and *Salmonella typhi*^13^,^14^,^15^,^16^,^17^,^18^. However, the effect of azithromycin on *S. maltophilia* biofilm formation is still not clearly defined, requiring further investigation.

In this study, we investigated biofilm formation by *S. maltophilia* using microtiter plate staining system plus scanning electron microscopy (SEM), and observed the *in vitro* effects of three FQs (moxifloxacin, levofloxacin, and ciprofloxacin) on different stages of biofilm formation for this organism. In addition, we investigated the effect of combined azithromycin and FQ treatment on the biofilm formation of *S. maltophilia*. Our study provides an experimental basis for possible clinical treatment of biofilm related infections caused by *S. maltophilia*.

## Results

### *In vitro* biofilm formation by *S. maltophilia*

**Figure 1:**
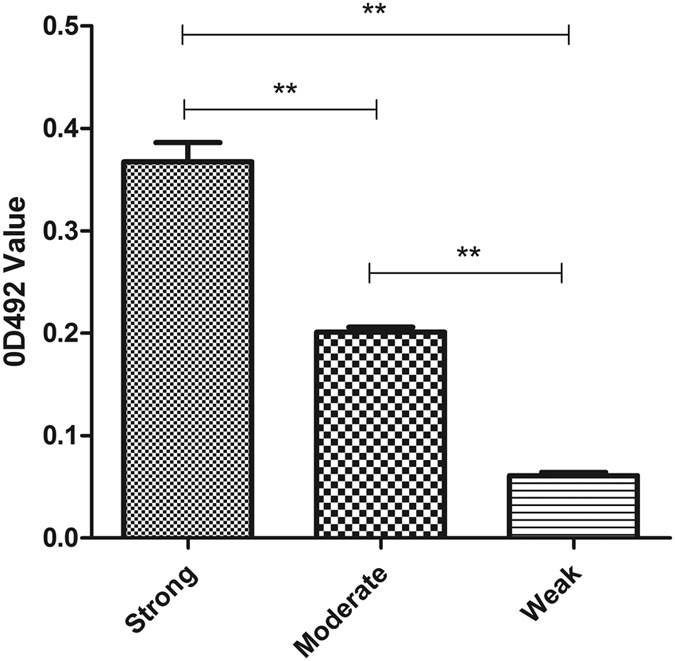
OD values of 45 *S. maltophilia* strains, indicating their capacity to form biofilms. Results are means ± SDs. **P < 0.01.

**Figure 2:**
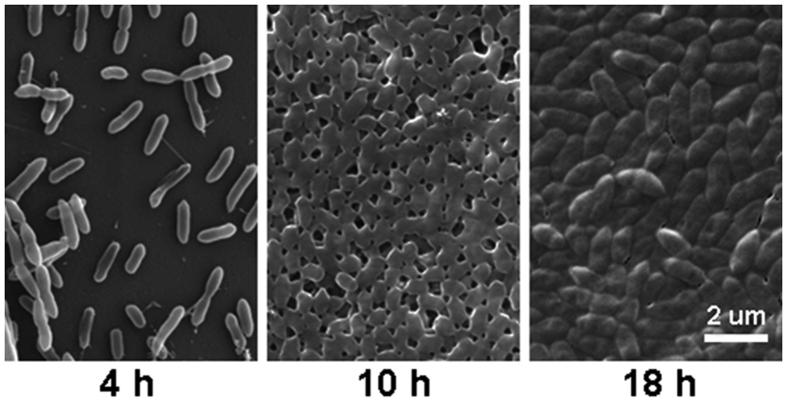
SEM images of one *S. maltophilia* strain* (10275) biofilms after 4 h (left), 10 h (middle) and 18 h (right).

Among 45 *S. maltophilia* strains studied, biofilm formation was weak in two (4.4%) strains, moderate in seven (15.6%) strains, and strong in 35 (77.8%) strains. There was no biofilm formation in one strain (Fig. 1). SEM analysis on biofilm formation by *S. maltophilia* strain 10275 (a strong biofilm producer randomly selected), showed that after 4 h incubation, the bacterial cells were clearly attached to the polystyrene surface and occupied approximately 30% of the surface. After 10 h of incubation, almost the entire polystyrene surface was occupied by cells, with little space in between attached cells. Bacterial cells began to secrete extracellular polymeric substances after 10 h, and completely filled the surface at 18 h (Fig. 2).

**Figure 3:**
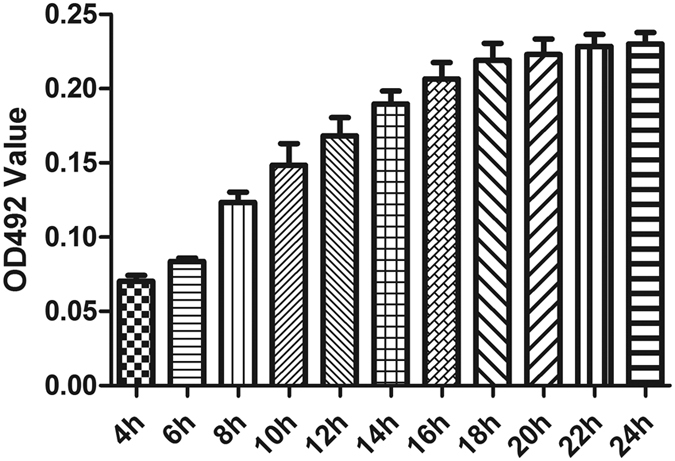
Average ODs at different time points (hours) of 15 randomly* selected *S. maltophilia* isolates. Results are means ± SDs. *Included 7 strong, 6 moderate and 2 weak biofilm producers.

To further understand the dynamics of biofilm formation, we measured optical density (OD) values at different time points (2 hourly) for 15 randomly selected *S. maltophilia* strains (7 strong, 6 moderate and 2 weak biofilm producers). The average OD values are shown in Fig. 3, and reveal that the organism was in the initial adhesive stage after 4 h of incubation, and in the exponential phase of growth after 8 h of incubation. Maximum growth was achieved after about 18 h of incubation (plateau phase).

### Antimicrobial susceptibility of planktonic and biofilm

A planktonic antimicrobial susceptibility test showed that approximately 90%, 80% and 70%, of the 45 *S. maltophilia* strains were susceptible to moxifloxacin, levofloxacin, and ciprofloxacin, respectively, and the results were consistent with previous studies^19^,^20^,^21^,^22^ (Table 1). We then compared the antibiotic susceptibility results between the planktonic and biofilm forms of 15 strains (7 strong, 6 moderate and 2 weak biofilm producers) randomly selected, for the antibiotics moxifloxacin, levofloxacin and ciprofloxacin. As shown in Table 2, the minimum inhibitory concentration (MIC) of the biofilm form (MIC-b) of each organism was much higher than that of the planktonic (MIC-p) form in all the 15 strains. Furthermore, the concentration of FQs required to inhibit re-growth of the biofilm cells was up to 256 times the MIC-b. These results indicate that the biofilms were more resistant to moxifloxacin, levofloxacin or ciprofloxacin than the planktonic culture (Table 3).

### Effects of fluoroquinolones (FQ) on *S. maltophilia* preformed biofilm of 15 randomly selected strains

**Figure 4:**
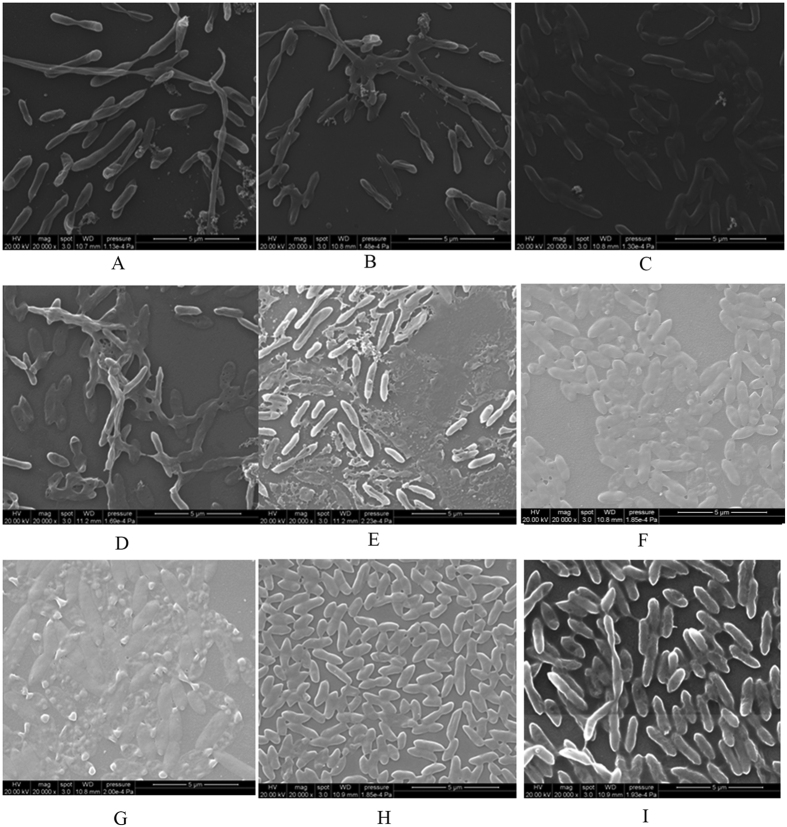
Scanning electron microscopy images of biofilms of one *S. maltophilia* strain (10275), treated with moxifloxacin for 4 h (A–C), 10 h (D–F) and 18 h (G–I). The concentrations of moxifloxacin were: 10 μg/mL (**A,D**,**G**), 50 μg/mL (**B,E**,**H**), 100 μg/mL (**C,F**,**I**). Magnification: ×20,000.

SEM was used to examine the effects of three different concentrations of moxifloxacin (10 μg/mL, 50 μg/mL and 100 μg/mL) on preformed biofilm of 15 randomly selected strains over three time points (4 hr, 10 hr and 18 hr). These three time points were selected based on findings from the 15 strains representing strong, moderate and weak biofilm producers (as previously mentioned), as well as the stages of biofilm production as determined by SEM (as mentioned above). Due to cost constrains, SEM was performed only on moxifloxacin, chosen because this FQ exhibited the most distinct inhibitory effect on *S. maltophilia* preformed biofilm as outlined below. Compared to the control (no antibiotic treatment, Fig. 2), moxifloxacin treatment significantly reduced the number of bacterial cells that adhered to the coverslip for the 4 hr, 10 hr and 18 hr incubation periods (Fig. 4). Moxifloxacin concentrations of 50 μg/mL or 100 μg/mL resulted in greater numbers of abnormal bacterial cells, especially at the early stages of biofilm formation (4 h). However, these biofilms were able to recover over time (Fig. 4).

**Figure 5:**
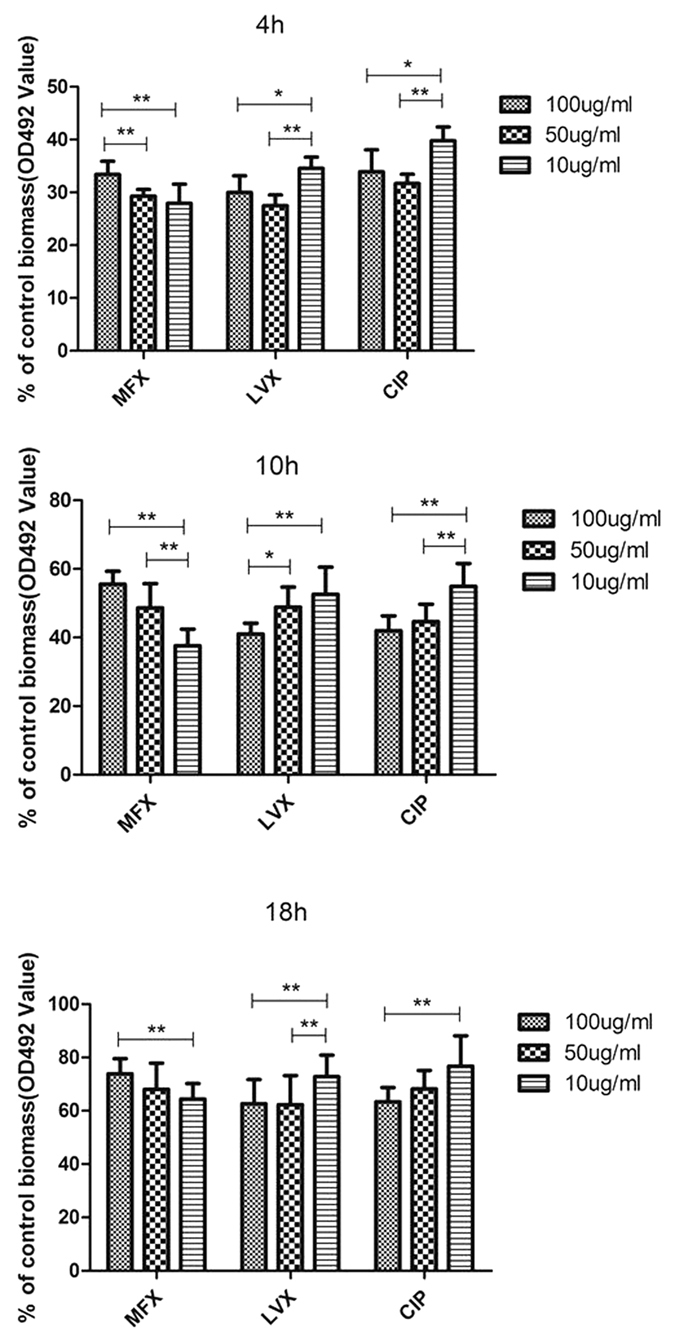
*In vitro* effects of fluoroquinolones on preformed biofilm of 15 *S. maltophilia* strains randomly selected. Results are means ± SDs. MXF: Moxifloxacin; LVX: Levofloxacin; CIP: Ciprofloxacin. *P < 0.05; **P < 0.01.

We further quantified, by measuring biofilm biomass, the effect of 3 different concentrations (10 μg/mL, 50 μg/mL and 100 μg/mL) of each of the three FQs (moxifloxacin, levofloxacin, ciprofloxacin), on preformed biofilm (biofilm allowed to form after 4 hr, 10 hr and 18 hr incubation before antibiotic addition) using 15 randomly selected *S. maltophilia* strains (7 strong, 6 moderate and 2 weak biofilm producers). As shown in Fig. 5, moxifloxacin treatment had a greater inhibitory effect on biofilm formation of immature preformed biofilms (4 h and 10 h) than mature preformed biofilms (18 h) as evidenced by significant differences in the biofilm biomass quantity. Specifically, the biomass for the immature (4 h or 10 h) preformed biofilm treated with moxifloxacin was only approximately 39% compared to that of untreated biofilm, but reached 69% for the mature (18 h) preformed biofilm treated with moxifloxacin. Similar results were observed following incubation with levofloxacin and ciprofloxacin (Fig. 5). All three FQs effectively inhibited biofilm formation (*P* < 0.05). However, in contrast to levofloxacin and ciprofloxacin, in which the level of inhibition of biofilm formation in preformed biofilm was more pronounced in the higher concentration (100 μg/mL), an opposite effect was observed for moxifloxacin. Specifically, the lower moxifloxacin concentration (10 and 50 μg/mL) produced higher inhibition levels for biofilm production in the 10 hr and mature (18 hr) preformed biofilms, than the 100 μg/mL concentration.

### *In vitro* effects of moxifloxacin on viability of *S. maltophilia* on preformed biofilms

**Figure 6:**
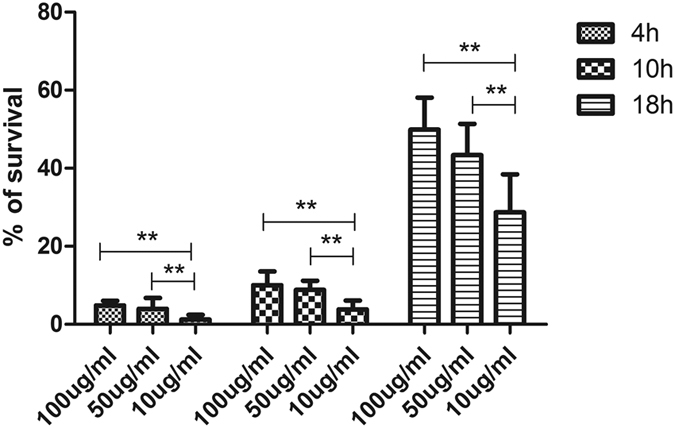
*In vitro* effects of moxifloxacin on viability of biofilms for 15 *S. maltophilia* strains randomly selected. **P < 0.01.

To further confirm our findings on the effect of moxifloxacin on biofilm formation using biomass measurements on preformed biofilms, we studied the effect of the 3 different moxifloxacin concentrations on the viability of 15 randomly selected *S. maltophilia* strains using a plate count technique^23^. As shown in Fig. 6, moxifloxacin (all concentrations) had a far greater effect on the viability of the organism in premature (biofilms formed after 4 h, 10 h incubation) (<10%) than in the mature (18 hr) biofilms (>28%) (P < 0.001). Furthermore, in both the premature and mature biofilms, the percentage survival of cells was greater in the higher concentration of the antibiotic. Overall, a greater proportion of cells survived (>28%) for the mature (18 hr preformed biofilms) than the immature (4 and 10 hr preformed biofilms) (<10%) (P < 0.001).

### Effects of a combination of FQs and azithromycin on *S. maltophilia* preformed biofilms

**Figure 7:**
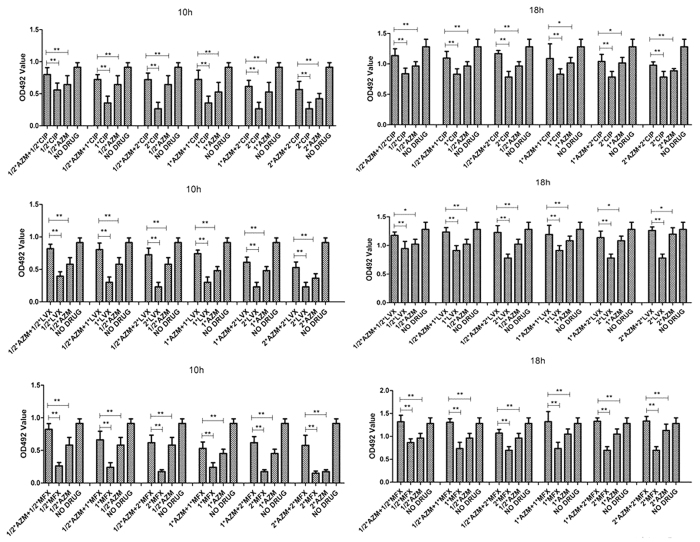
*In vitro* effects of fluoroquinolones combined with azithromycin on biofilms of 15 randomly selected *S. maltophilia* strains after 10 h or 18 h. Results are means ± SDs. MXF: Moxifloxacin; LVX: Levofloxacin; CIP: Ciprofloxacin; AZM: azithromycin. *P < 0.05; **P < 0.01.

To further examine the effects of antibiotics on biofilm formation by *S. maltophilia*, a combination of FQs and azithromycin was tested on the 15 randomly selected *S. maltophilia* strains. Interestingly, compared with FQs or azithromycin alone, the combination of FQs and azithromycin reduced the inhibiting effect on *S. maltophilia* preformed biofilms (*P* < 0.05). We tested several combinations of different drug concentrations, and none of the combinations increased the associated inhibition (Fig. 7). The worst inhibiting effect was observed when azithromycin was combined with moxifloxacin (Fig. 7).

### Effect of a combination of ciprofloxacin and azithromycin on preformed biofilm of *Pseudomonas aeruginosa*

**Figure 8:**
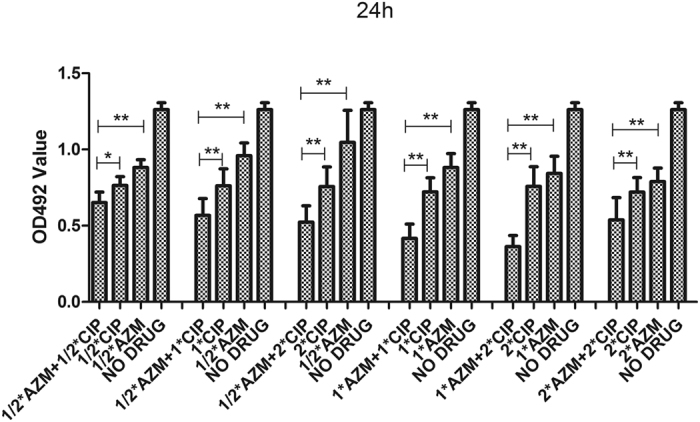
*In vitro* effects of ciprofloxacin combined with azithromycin on *Pseudomonas aeruginosa* biofilms after 24 h. Results are means ± SDs. CIP: Ciprofloxacin; AZM: azithromycin. *P < 0.05; **P < 0.01.

Several studies have indicated that a combination of FQs and azithromycin has a synergetic effect on *Pseudomonas aeruginosa* biofilm^13^,^14^. To validate our findings regarding the antagonistic effect of combined FQs and azithromycin on *S. maltophilia*, we also tested the effect of a combination of ciprofloxacin and azithromycin on 15 randomly selected *Pseudomonas aeruginosa* biofilm^14^. As depicted in Fig. 8, our results confirm the previously reported antagonistic effect.

## Discussion

Biofilms are products of bacterial adherence to a natural or living surface. The starting point of various chronic infections is often the formation of a biofilm by the infecting organism. Thus targeting the biofilm phase of the organism is an important consideration for effective treatment of most chronic infections. Selection of antibiotics to treat *S. maltophilia* infections is often based on conventional antimicrobial susceptibility results of organisms grown planktonically in liquid media. However, it is known that organisms such as *S. maltophilia* actually grow as biofilms which exhibit higher resistance to antibiotics compared to floating bacteria on epithelial cells of the airway. This suggests that selection of antibiotics for *S. maltophilia* infections based on biofilm susceptibility testing may provide an accurate and effective guide on appropriate treatment^11^. Consequently, it is critical to understand the dynamics of biofilm formation by *S. maltophilia* which could give insights into the treatment of infections caused by this organism.

In this study, using a microplate assay, we demonstrated the ability of the majority of *S. maltophilia* clinical strains studied to adhere to abiotic surface, which is in agreement to previous studies^8^,^24^,^25^. Furthermore, using scanning electron microscopy (SEM) and microplate plate assay, we observed dynamic biofilm formation of *S. maltophilia* in vitro, including initial attachment of the organism to polystyrene surface after 4 hrs incubation, and achieving maximal biofilm growth after approximately 24 hrs.

*S. maltophilia* is naturally resistant to many antimicrobial agents. In this study, we demonstrated that *S. maltophilia* biofilms are more resistant to three fluoroquinolone (FQ) antibiotics, which is consistent with previous results^21^,^26^. Thus caution must be exercised when treating *S. maltophilia* infections based on antibiotic susceptibility testing of planktonic forms of the organism. The enhanced resistance to antimicrobial agents by *S. maltophilia* is due to several factors, including chromosomally encoded multidrug resistance, efflux pump and antibiotic modifying enzymes^24^.

It is widely believed that sub-MICs of FQs have inhibitory effects on bacterial adhesion^12^,^25^,^27^. It is thus possible that this class of antibiotics may also have inhibitory effects on *S. maltophilia* biofilm formation^12^, and this inhibition may be dose-dependent^25^. Based on our findings showing that *S. maltophilia* is in the initial adhesive stage at 4–6 h of incubation, exponential phase of biofilm formation at 10–16 h, and forms mature biofilm at 18–24 h, we chose the 4, 10 and 18 h incubation periods for further study of this organism.

The three FQs studied (moxifloxacin, levofloxacin, and ciprofloxacin) each at 3 different concentrations (10, 50, 100 μg/ml), exhibited greater inhibitory effects on preformed *S. maltophilia* non-mature biofilms (when FQs were applied at 4 h or 10 h incubations). However, the inhibitory effect was significantly reduced for mature biofilms (when FQs were applied on biofilms formed after 18 h incubation). These findings which were confirmed by SEM (Fig. 4), may be explained by differences in antibiotic permeability of the biofilm in the early and mature stages of development, heterogeneity associated with cell growth, quorum sensing systems, and other mechanisms^9^. Thus based on our findings, early identification (and treatment) of organisms causing infections may be important for controlling biofilm formation.

Interestingly, for mature preformed biofilms (18 h), the inhibitory effect of moxifloxacin at 10 μg/mL (and 50 μg/mL) concentration was surprisingly even greater than at 100 μg/mL. This is consistent with a previous report that showed that the inhibitory effect of moxifloxacin on biofilms is different from that of other FQs^25^. It is not clear however, why lower concentrations of moxifloxacin has a greater inhibitory effect than higher concentrations. Our results suggest that the conventional dose of moxifloxacin (400 mg, qd), which is equivalent to 10 μg/mL in human alveolar lining fluid, can be used as an alternative treatment for *S. maltophilia* biofilm-related lung infection or colonization.

Although the effect of a combination of FQs and azithromycinon on biofilm formation has been studied previously^24^, results have been mixed. Specifically, this combination therapy has been shown to have a synergistic effect on *Pseudomonas aeruginosa* and *Legionella* biofilm formation, but an antagonistic effect on *Mycobacterium avium* and *Salmonella typhi*^13^,^14^,^15^,^16^,^17^. In this study, we found that azithromycin somehow weakened the antibacterial effect of FQs, especially when moxifloxacin was involved. We speculate that azithromycin may interfere with FQs, resulting in a reduction in their antibacterial effectiveness against some bacteria.

Previous studies have also reported that clarithromycin reduces the effects of gatifloxacin and levofloxacin on *Mycobacterium avium*. This may be due to clarithromycin-induced inhibition of protein synthesis interfering with the bactericidal activity of FQs^28^. A similar mechanism may be involved with azithromycin. Wang *et al*.^29^ also reported that *ica* (intercellular adhesin) operon encoded enzymes may mediate intercellular adherence of bacteria and the accumulation of multilayer biofilms through the production of polysaccharides that promote intercellular adhesion. A sub-inhibitory concentration of erythromycin has been shown to induce *ica* expression, suggesting a possible role for macrolides in biofilm formation^29^. Further studies are required to determine whether a combination of moxifloxacin and azithromycin has a similar mechanism of action.

This study has several limitations. First, due to cost implications, we mainly relied on biomass measurement (spectrophotometric microtiter assay) for biofilm quantification, and this method measures both viable and unviable bacteria, including the extracellular polymeric substance. However, in agreement with several studies^12^,^30^, we demonstrated in one experiment the high correlation between the microtiter plate assay (OD readings) and the viable cell enumerations (plate count) in the measurement of biofilm production by bacteria.

Second, most of the assays to examine the effects of the 3 FQs on biofilm formation were performed on a limited number of isolates (15; 7 strong, 6 moderate and 2 weak biofilm producers) for the biofilm assay and 3 time periods (4 h, 10 h and 18 hr incubation periods). It can be argued that the small number of isolates used and the selected time points, may not be representative enough to give sufficient statistical confidence to the findings. However, the time points selected were based on our findings involving 15 strains chosen randomly to represent strong, moderate and weak biofilm producers, as well as SEM analysis showing different stages of biofilm formation. Furthermore, SEM and the biofilm viability plate assay were performed on 15 isolates only. It is arguable that studies based on different *S. maltophilia* strains and on higher numbers of isolates would yield different results. However, our findings are in agreement with previous studies, and give some insight into biofilm formation by *S. maltophilia* in the presence of a selected number of FQs. Nevertheless, we consider our findings as exploratory and hypothesis generating, requiring confirmation in the future.

Finally, we didn’t study in detail the suppressive effects of levofloxacin and ciprofloxacin on preformed biofilm and also did not explore in-depth the effects of azithromycin on biofilm formation, as the devices commonly used for SEM study of biofilms are expensive.

In conclusion, findings from this study suggest that early use of clinically acceptable concentrations of selected FQs (10 μg/mL), especially for moxifloxacin, can inhibit biofilm formation by *S. maltophilia.* Our study provides an experimental basis for a possible optimal treatment strategy for biofilm-related infections caused by *S. maltophilia*.

## Materials and Methods

### Strains and antibiotics

Forty-five *S. maltophilia* strains were collected and stored in our laboratory as previously described^31^. Antimicrobial drugs (including azithromycin, moxifloxacin, levofloxacin and ciprofloxacin) ordered from the National Institute for the Control of Pharmaceutical and Biological Products, Beijing, or Bayer company, Germany, were used. *Pseudomonas aeruginosa* ATCC 27853 was used for quality control in all experiments.

### Scanning electron microscopy (SEM) observation of biofilm formation

SEM was used to observe biofilm formation and effects of antimicrobial drugs on biofilm formation^12^. In order to observe the morphological changes associated with biofilms following moxifloxacin treatment, biofilms were allowed to grow on sterile flat-bottom 6-well polystyrene tissue culture plates (LabServ, Thermo Fisher Scientific, USA) in the presence or absence of moxifloxacin treatment. Fresh Cation-Adjusted Mueller Hinton Broth (CAMHB) without any antibiotic was used as a control. The samples were fixed with 2.5% glutaraldehyde for at least 4 h at 4 °C. After washing with phosphate buffered saline (PBS), the samples were dehydrated in a series of ethanol solutions of increasing concentration (50 to 100%). The cells were subsequently washed with pure acetone, followed by isoamyl acetate. They were then dried with a critical point dryer (Hitachi, Japan). The dried samples were coated with 15-nm gold using an argon automatic sputter coater (Hitachi, Japan). After processing, the samples were viewed with a Philips XL30CP scanning electron microscope.

### Biofilm formation assay

*S. maltophilia* isolates (n = 45) and *S. maltophilia* ATCC13637 were used in the biofilm formation assay. Microplate assays were performed as previously described with some modifications^12^,^32^,^33^. Briefly, overnight cultures of *S. maltophilia* were standardized to a 0.5 MacFarland standard (equivalent to 1.5 × 10^8^ CFU/mL), and then diluted (1:100) with fresh Luria Bertani broth. Aliquots (200 μL) of standardized inocula were added to the wells of sterile flat-bottom 96-well polystyrene tissue culture plates (Thermo Fisher Scientific, USA) and incubated at 37 °C over a series of time-points (2, 4, 6, 8, 10, 12, 14, 16, 18, 20, 22, and 24 h) in a closed and humidified plastic container. The medium was then discarded and non-adherent cells were removed by washing three times in sterilized ultrapure water. The cells were stained with 0.01% (w/v) crystal violet for 20 min. The excess stain was then removed by washing with water and the stained biofilms were dried for 30 min at ambient room temperature and extracted with 33% (v/v) glacial acetic acid. The amount of biofilm produced was quantified by measuring the optical density at 492 nm (OD_492_) using a plate reader (Varioskan Flash; Thermo Fisher Scientific, USA). Non-inoculated media were used as a control. The low cut-off point for biofilm production was chosen according to the criteria described by Christensen *et al*.^34^, i.e. the cut-off point was defined as three standard deviations above the mean optical density of the control (ODc) wells^12^. Based on this cut-off, strains were classified into the following categories: no biofilm producer (OD ≤ ODc), weak biofilm producer (ODc < OD ≤ 2 × ODc), moderate biofilm producer (2 × ODc < OD ≤ 4 × ODc), and strong biofilm producer (4 × ODc < OD)^31^. Each isolate was assayed three times and the results were presented as the average of the three assays.

### Planktonic antimicrobial susceptibility test

The minimum inhibitory concentrations (MICs) of moxifloxacin, levofloxacin, ciprofloxacin were determined by the CLSI broth microdilution technique (M100-S22)^35^. *Escherichia coli* ATCC 25922 was used as the quality control strain.

### Biofilm antimicrobial susceptibility test for 15 randomly selected strains

The minimum inhibitory concentration in the biofilm plate (MIC-b) was defined as the lowest concentration of an antibiotic in which a planktonic bacterial population could not be established by shedding of bacteria from the biofilm^23^. MIC-b was assessed as previously described, with minor modifications. In brief, aliquots (75 μL) of the standardized inocula were added to the wells of a 96-well microplate and incubated for 24 h at 37 °C for production of biofilm. The medium was then discarded, wells washed with sterilized saline water, and 100 μL of 1:2 dilutions of moxifloxacin, levofloxacin or ciprofloxacin (from 2048 μg/mL to 0.50 μg/mL) prepared in fresh Mueller Hinton (MH) broth, was added to the established biofilms. The 96-well plates were wrapped with aluminum foil and placed in an incubator at 37 °C for 18–20 h. The MIC-b was determined as the minimum antibiotic concentration in which a planktonic bacterial population could not be established by shedding of bacteria from the biofilm. All samples were assessed in quadruplicate.

### Determination of minimum regrowth concentration (MRC)

MRC, which is defined as the minimum antibiotic concentration (μg/ml) required to inhibit regrowth of the cells, was determined as previously described^36^. Briefly, 100 μL of appropriate dilutions of each of the three FQs in MH broth, were transferred into the 96-well tissue culture microtiter plate wells with established biofilms and incubated for 18–20 h at 37 °C. Each well was washed three times with PBS under aseptic conditions, 100 μL TSB added, and the samples were incubated for 24 h at 37 °C. Minimum antibiotic concentration which inhibited regrowth of the cells (mg/L) was determined.

### Determination of minimum biofilm eradication concentration (MBEC)

MBEC was defined as the lowest antimicrobial concentration which prevented bacteria regrowth after antimicrobial exposure. In other words, the lowest antibiotic concentration required to eradicate the biofilm. This assay was performed on 15 randomly selected strains. The MBEC assay was performed as previously described^37^, with slight modifications. The 24 h biofilms in a 96-well tissue culture microtiter plate were washed three times with 250 μL PBS and air dried. Serial 2-fold dilutions ranging from 2048 to 0.5 μg/ml for LVX and CIP, and from 1024 to 0.25 μg/ml for MFX, were prepared in CAMHB. A 200 μL sample of each concentration for each antibiotic was added to a corresponding well, and plates were incubated for 24 h at 37 °C. After the incubation, antibiotic solutions were aspirated gently, plates were washed two times with sterile PBS solution, and wells were scraped thoroughly, with particular attention for the well edges. Well contents were transferred to 1 ml of PBS solution and placed in a sonicating water bath (Bandelin sonopuls HD 2200) for 5 min to disrupt the biofilm, and 100 μL samples were plated on TSA. Colonies were counted after 24 h at 37 °C. The MBEC was defined as the lowest concentration of antibiotic that prevented bacterial regrowth.

### Effects of FQs on *S. maltophilia* preformed biofilm

This test, which was done on 15 randomly selected strains (7 strong, 6 moderate and 2 weak biofilm producers), was performed as previously described with minor modifications^12^. Biofilm was allowed to form after 4 h, 10 h, and 18 h incubation periods (which were defined as the preformed biofilm) at 37 °C. After this, the supernatant from each well was gently discarded, and wells washed gently three times with sterile saline water. Different concentrations of moxifloxacin, levofloxacin or ciprofloxacin (100, 50 and 10 μg/mL) prepared in 200 μL of fresh CAMHB was then added to the wells. Fresh CAMHB without any antibiotic was used as a control. The plates were incubated at 37 °C for 6 h, and the quantity of biofilm produced determined as previously described at OD_492_.

### Effects of moxifloxacin on viability of *S. maltophilia* on preformed biofilms

This test was performed as previously described with minor modifications^23^. The viability of the biofilm was determined by plate counts and was performed on 15 randomly selected strains. Following the effect of different concentrations of moxifloxacin (100, 50 and 10 μg/mL) on the *S. maltophilia* preformed biofilm allowed to form after 4 h, 10 h, and 18 h incubation periods, supernatant was discarded, the wells were washed and the surfaces of the wells were scraped with sterile cotton swabs (three wells per dilution). Swabs were transferred into tubes containing 2 mL of PBS, sonicated (Transonic SB-120DT, Shanghai, China) for 3 min, and vortexed vigorously to aid dissolution of bacterial clumps. The number of viable cells was estimated by plating serial dilutions of these suspensions on Mueller Hinton agar (Oxoid Ltd.) plates.

### Effect of combined FQs and azithromycin on *S. maltophilia* preformed biofilm

This test was performed on the 15 randomly selected *S. maltophilia* strains and *Pseudomonas aeruginosa* strains. After 10 and 18 h (for *S. maltophilia* strains) and 24 h (for *Pseudomonas aeruginosa* strains) incubation periods at 37 °C, the supernatant from each well was gently discarded. Each well was then washed three times with sterile saline water without destroying the attached biofilm. Different concentrations of moxifloxacin, levofloxacin or ciprofloxacin (0.5×, 1× or 2× of the MIC) were prepared following dilution in 100 μL of fresh CAMHB in the wells of the micro-titer plate. Additionally, various concentrations of azithromycin (0.5×, 1×, or 2× of the MIC) were added to wells on the same plate. Fresh CAMHB without antibiotics was added to the control wells. The plates were incubated at 37 °C for 6 h and the OD_492_ was measured.

### Statistical analysis

All assays were performed in triplicate (at a minimum) and repeated three times. Multiple comparisons of responses to antibiotics and the inhibition of biofilm formation were performed using multiple factor variance analysis. A P-value < 0.05 was considered statistically significant. Statistical analysis was conducted with SPSS Statistics 17.0 software.

## Additional Information

**How to cite this article**: Wang, A. *et al*. Effects of Fluoroquinolones and Azithromycin on Biofilm Formation of *Stenotrophomonas maltophilia*. *Sci. Rep.* **6**, 29701; doi: 10.1038/srep29701 (2016).

## Figures and Tables

**Table 1:** Planktonic antimicrobial susceptibility of 45 *S. maltophilia* isolates.

	MIC Range	Mode	MIC50^a^	MIC90^b^	%^c^
**Moxifloxacin**	0.03125–8	0.25	0.25	4	88.64
**Levofloxacin**	0.0625– > 8	0.25	0.5	4	79.55
**Ciprofloxacin**	0.5– > 16	1	1	16	65.91

^a^MIC50, MIC inhibiting 50% of the isolates tested.

^b^MIC90, MIC inhibiting 90% of the isolates tested.

^c^Percentages of susceptibility were calculated on the basis of interpretative breakpoints suggested by CLSI guidelines^35^.

**Table 2:** Comparison of antimicrobial susceptibility between planktonic and biofilm forms of the organism among 15 randomly selected *S. maltophilia* isolates.

ID of isolates	MIC-p^a^			MIC-b^b^		
	MXF	LVX	CIP	MXF	LVX	CIP
2443	0.5	0.5	1	2	>2048	32
10275	0.5	2	2	>1024	>2048	>2048
2322	4	8	16	>1024	>2048	>2048
7225	0.0625	0.5	2	2	16	32
2782	4	8	16	16	32	32
2618	0.5	2	4	16	32	32
6414	0.0625	0.25	1	1	4	32
7387	0.25	0.5	1	1	2	2
7249	0.0625	0.5	2	2	8	32
11252	0.25	0.0625	1	4	16	32
11829	0.25	0.5	1	4	16	32
2579	0.125	0.25	1	4	32	32
283	0.25	0.5	2	4	16	16
2629	2	4	8	128	16	32
11757	0.25	0.0625	0.5	0.5	2	2

MIC-p^a^, minimum inhibitory concentration of floating bacteria.

MIC-b^b^, minimum inhibitory concentration in the biofilm plate.

Values represent the geometric mean of three independent experiments.

MXF: Moxifloxacin; LVX: Levofloxacin; CIP: Ciprofloxacin.

**Table 3:** Comparison of antimicrobial susceptibility between MRC and MBEC among 15 randomly selected *S. maltophilia* isolates.

ID of isolates	MRC^a^ (μg/ml)			MBEC^b^(μg/ml)		
	MXF	LVX	CIP	MXF	LVX	CIP
2443	>1024	>2048	>2048	>1024	>2048	>2048
10275	>1024	>2048	>2048	>1024	>2048	>2048
2322	>1024	>2048	>2048	>1024	>2048	>2048
7225	>1024	>2048	2048	>1024	>2048	>2048
2782	1024	>2048	>2048	>1024	>2048	>2048
2618	1024	>2048	>2048	>1024	>2048	>2048
6414	1024	>2048	>2048	>1024	>2048	>2048
7387	1024	>2048	2048	>1024	>2048	>2048
7249	>1024	>2048	>2048	>1024	2048	>2048
11252	>1024	>2048	2048	>1024	>2048	1024
11829	>1024	>2048	>2048	>1024	>2048	2048
2579	1024	>2048	2048	>1024	>2048	>2048
283	>1024	>2048	>2048	>1024	2048	>2048
2629	>1024	>2048	>2048	>1024	>2048	>2048
11757	>1024	>2048	>2048	>1024	>2048	2048

MRC^a^: minimum regrowth concentration.

MBEC^b^: minimum biofilm eradication concentration.

MXF: Moxifloxacin; LVX: Levofloxacin; CIP: Ciprofloxacin.
